# Behavioral and Psychological Symptoms of Dementia as a Means of Communication: Considerations for Reducing Stigma and Promoting Person-Centered Care

**DOI:** 10.3389/fpsyg.2022.875246

**Published:** 2022-03-29

**Authors:** Alison Warren

**Affiliations:** The Department of Clinical Research and Leadership, George Washington University School of Medicine and Health Sciences, Washington, DC, United States

**Keywords:** Alzheimer’s disease, dementia, BPSD, unmet needs, personhood, stigma

## Abstract

Dementia has rapidly become a major global health crisis. As the aging population continues to increase, the burden increases commensurately on both individual and societal levels. The behavioral and psychological symptoms of dementia (BPSD) are a prominent clinical feature of Alzheimer’s disease and related dementias (ADRD). BPSD represent a myriad of manifestations that can create significant challenges for persons living with dementia and their care providers. As such, BPSD can result in detriments to social interaction with others, resulting in harm to the psychosocial health of the person with dementia. While brain deterioration can contribute to BPSD as the disease progresses, it may be confounded by language and communication difficulties associated with ADRD. Indeed, when a person with dementia cannot effectively communicate their needs, including basic needs such as hunger or toileting, nor symptoms of pain or discomfort, it may manifest as BPSD. In this way, a person with dementia may be attempting to communicate with what little resources are available to them in the form of emotional expression. Failing to recognize unmet needs compromises care and can reduce quality of life. Moreover, failing to fulfill said needs can also deteriorate communication and social bonds with loved ones and caregivers. The aim of this review is to bring the differential of unmet needs to the forefront of BPSD interpretation for both formal and informal caregivers. The overarching goal is to provide evidence to reframe the approach with which caregivers view the manifestations of BPSD to ensure quality of care for persons with dementia. Understanding that BPSD may, in fact, be attempts to communicate unmet needs in persons with dementia may facilitate clinical care decisions, promote quality of life, reduce stigma, and foster positive communications.

## Introduction

As the most common form of dementia, Alzheimer’s disease (AD) affects over 6 million individuals aged 65 and older in the United States alone (Alzheimer’s Association, 2021). Worldwide, nearly 50 million people are affected by AD and other dementias and this number is projected to continue to double every 20 years (Grande et al., 2020). Despite this widespread prevalence, pathophysiology is still poorly understood (Bottero et al., 2021) and neuronal destruction “cannot be prevented, slowed, or cured” (Leandrou et al., 2020, p.176).

The most salient features of AD include progressive declines in memory, language, cognitive functions, and activities of daily living; death ultimately ensues (Leandrou et al., 2020). Furthermore, these neurocognitive declines are concomitant with behavioral and psychological symptoms of dementia (BPSD), such as depression, anxiety, apathy, and agitation (Peña-Casanova et al., 2012; Stella et al., 2015). Another feature of cognitive decline that can be quite disconcerting and disorienting in persons with dementia is topographical disorientation, characterized by deterioration of spatial and navigational abilities (Lopez et al., 2018). This loss of spatial orientation, which may occur in transient episodes, can have a profound effect on the autonomy and daily activities of persons with dementia (Caffò et al., 2020). As these symptoms fluctuate throughout the continuum of the disease, aspects of cognition, emotion, memory, and perception are maintained (Sabat, 2018; Gazzaniga et al., 2019), the experiences of which may imbue inordinate struggle to effectively convey to caregivers and loved ones (Swall et al., 2017). The conundrum then, lies within the dilemma of labeling these behaviors “BPSD,” thereby medicalizing, and possibly stigmatizing the individual, resulting in neglection of the thoughts and emotions the individual is attempting to convey. Indeed, considering the profound language and cognitive difficulties faced by persons with dementia (PWD), it is likely that some manifestations of BPSD are, in fact, a means through which to communicate.

## Background

Behavioral and psychological symptoms of dementia are a major clinical feature of AD affecting patients, family, and caregivers (Holmgren et al., 2014). BPSD can include depression, anxiety, and apathy initially, and often progresses to irritability, agitation, disinhibition, delusions, and hallucinations as the disease becomes progressively more severe (Peña-Casanova et al., 2012; Chakraborty et al., 2019). Alterations in motor behavior (i.e., wandering), sleep, and nighttime behavior may also be present (Kim et al., 2021). Medial temporal lobe atrophy, specifically the hippocampus, seems to be associated with BPSD etiology (García-Alberca et al., 2019). Neuropsychiatric symptoms are often expressed in later stages of AD but can occur at any stage, consistent with serotonergic system alterations (Chakraborty et al., 2019), neuroinflammation, and impairments in cholinergic function in the cortex, hippocampus, and limbic system (Holmgren et al., 2014). The pervasive effects of BPSD in patients and caregivers notwithstanding, there are no FDA-approved drugs to treat BPSD in the United States (Chakraborty et al., 2019).

While estimates vary, meta-analysis suggests a prevalence of approximately 50% for the most common BPSD symptoms (Sutin et al., 2021). Evidence suggests that personality traits may serve as risk and protective factors in the development of BPSD. Specifically, premorbid neuroticism has been observed to be significantly associated with BPSD development (Young et al., 2019); Young et al. (2019) further found evidence for protective premorbid personality traits including conscientiousness, extraversion, openness and agreeableness. Purpose in life has also been identified as protective factor, strongly associated with resilience to the psychological symptoms of BPSD (i.e., depression, confusion, uncontrolled temper), but was not shown to be protective against motor or perceptual symptoms seen in BPSD (Sutin et al., 2021).

### The Problem

Behavioral and psychological symptoms of dementia represent a major burden to patients and caregivers, along with significant challenges to quality of care in the most well-intentioned caregivers (Honda et al., 2018). Furthermore, BPSD have been associated with decreased functional abilities; poorer prognosis; increased caregiver burden (Brodaty et al., 2003); premature nursing home placement (Whall and Kolanowski, 2004; Cunningham et al., 2019); reductions in quality of life; accelerated cognitive decline; and higher cost of care (García-Alberca et al., 2019). Barriers in communication between persons with AD and caregivers exacerbates what is already a devastating disease to those experiencing and witnessing its duration. In fact, caregiver distress has been associated with increases in manifestations of BPSD, patient abuse, and odds of institutionalization of the patient (Peterson et al., 2016). This bidirectional relationship between caregiver burden and neuropsychiatric symptoms is a key factor in quality-of-life outcomes in the caregiver-patient dyad (Isik et al., 2019). As verbal communication abilities worsen in persons with AD, emotions remain a key element of communication and are preserved well into the late stages of AD (Lee et al., 2019). The retained emotional component in the person with AD creates a “heightened tendency to take on the emotions of those around them,” which, when negative, can manifest as BPSD (Fredericks et al., 2018, p. 471). Considering the preserved emotions and cognitions in persons with AD who ultimately lose the ability to effectively convey said feelings and thoughts, an examination of BPSD as a means of communication is warranted. Finally, the term “BPSD” itself may contribute to the stigma connected with Alzheimer’s Disease and related dementias (ADRD), and thereby pathologize emotions, behavior, and communication attempts (Markwell, 2016; Cunningham et al., 2019). The purpose of this article therefore aims to increase awareness and understanding of unmet needs in the context of BPSD, to thereby improve quality communications with caregivers. Doing so may aid in increasing quality of life in persons with dementia by ensuring their needs are met, they are treated with dignity by respecting their personhood, and fostering positive communications.

## Methodology

This is a review of the literature utilizing a person-centered lens to evaluate additional causes of BPSD manifestations, increase caregiver awareness of unmet needs, and foster positive communication and increased quality of life in persons with dementia. A search limited to peer-reviewed articles was conducted to include the last decade, from 2012 and up to and including February 2022 through the George Washington University Health Sciences Library Catalogue (Himmelfarb), the Harvard Library Catalogue (HOLLIS), Google Scholar, PubMed, and PsychINFO databases. Topics included Alzheimer’s disease; dementia; gerontology; formal and informal caregivers; caregiver burden; BPSD; unmet needs; stigma; and personhood. Exclusion criteria included dementias with very characteristic personality changes due to neuronal detioration, including Frontotemporal dementia, Lewy Body dementia, Vascular dementia, and Korsakoff syndrome. The main keywords chosen in this search included “Alzheimer’s disease” and “dementia” and the following inclusive AND keywords “BPSD”; “unmet needs”; “stigma”; and “communication.” In addition to the above-mentioned databases, snowballing techniques were used in several salient and controversial papers related to the topic. In a survey, 329 papers were initially identified. Articles that focused on caregiver burden, pharmacological intervention, and comorbidities were further excluded. Systematic reviews and meta-analyses were prioritized where available, along with prioritization of the most recent relevant articles, resulting in 14 total studies selected for review. These person-centered care studies were further grouped into the effect of person-centered care on BPSD, BPSD as manifestations of unmet needs, language and attitudes contributing to BPSD and stigma associated with AD, and non-pharmacological interventions for BPSD.

### Person-Centered Care and Behavioral and Psychological Symptoms of Dementia

In the 1980s, a well-respected psycho-gerontologist, Tom Kitwood, pioneered a seminal paradigm shift in the approach to dementia care and the understanding of dementia behavior (Kitwood, 1988; Cunningham et al., 2019). This approach reframes the individual with dementia “as a person in the fullest possible sense: he or she is still an agent, one who can make things happen in the world, a sentient, relational, and historical being” (Kitwood, 1993, p. 541). He maintained that much of what we consider BPSD are in fact, “valid responses to inappropriate external circumstances and relational approaches,” suggesting the need for greater focus on the behaviors of family members and caregivers (Cunningham et al., 2019, p. 1009). Kitwood brought concepts of personhood to the forefront, along with the social behavior tendencies of caregivers and loved ones that, while often unintentional, are “highly damaging from the recipient’s point of view” (e.g., treachery; disempowerment; infantilization; condemnation; intimidation; stigmatization; outpacing, invalidation; banishment; objectification) (Kitwood, 1993, p. 542). These behaviors were collectively termed “malignant social psychology” (MSP), denoting behaviors that amount to treating these individuals as less than human (Cunningham et al., 2019). To counter MSP, twelve indicators of positive person work were operationalized, including “assertion of desire, emotional ambience, initiation of social contact, showing affection, sensitivity to others’ feelings, self-respect, acceptance of other dementia sufferers, humor, creativity, helpfulness, taking pleasure, and physical relaxation” (Kitwood, 1993, p. 542; Cunningham et al., 2019). These indicators also serve to further highlight avenues with which PWD retain and communicate *via* preserved emotions and cognitions. The expression of emotions by these individuals, who endure language and cognitive difficulties, are a key element in the assessment of needs (i.e., pain, hunger, thirst), that are the fundamental elements of PCC (Lee et al., 2019). Indeed, emotional expression, both positive and negative, may serve as an essential representation of an individual’s needs and feelings, thereby facilitating PCC (Lee et al., 2019).

Kitwood’s framework of PCC has persisted as a critical component in caring for persons with ADRD, particularly in long-term care facilities. Terkelsen et al. (2020) reviewed 154 studies investigating the experiences of dementia patients and formal caregivers’ use of Kitwood’s framework and found overall positive experiences achieved by its implementation. While the vast majority of these studies suggested PCC to be beneficial in practice (Terkelsen et al., 2020), practically there is a lack of research on PCC implementation into nursing home care (Boersma et al., 2019). A study by Kolanowski et al. (2015) examined how nursing home staff obtain necessary PCC information utilized for PWD who exhibit BPSD. They noted nursing homes that implement this culture change reflective of PCC demonstrate significant positive outcomes for both residents and staff, but adoption is slow, in part, due to communication breakdown and interference in information exchange (Kolanowski et al., 2015). A recent adaptation of PCC, the Veder contact method (VCM), an emotion-oriented care model, was studied in the setting of a 24-h residential care facility by Boersma et al. (2019). Significant improvements were observed in caregiver communication, positive interactions, and resident behavior and quality of life (Boersma et al., 2019). These studies highlight several strengths inherent in PCC approaches. They demonstrate the powerful impact of PCC on caregiver communication and quality of life in PWD, even with short-term implementation. Limitations of these studies are similar to many other studies in PWD residing in long-term care facilities, including but not limited to high turnover rates, time investiment necessary for education and training, and post-study long-term compliance. Moreover, as many of these studies emphasize more qualitative collections of evidence, there are variations in methodological quality assessment.

### Behavioral and Psychological Symptoms of Dementia as Attempts to Communicate Unmet Needs

Behavioral and psychological symptoms of dementia may represent attempts to communicate unmet needs in persons with ADRD, who either lack the ability to effectively communicate or fulfill these needs themselves. Particularly in this patient population, emotional expression often signifies underlying needs and feelings, the recognition of which is crucial to implementation of person-centered care (Lee et al., 2019). Optimizing their preserved emotional capacities may “help AD patients to compensate for, and resiliently adapt to the impairment of cognitive functions caused by the disease” (Zhang et al., 2015, p. 208).

Unfortunately, many regard the BPSD as “disruptive,” rather than a meaningful expression of a need or pursuit of a goal, and thereby also miss opportunities to intervene and even prevent BPSD (Algase et al., 1996). Reframing BPSD to include this consideration can serve to shift the perspective of a constellation of behaviors and symptoms that many consider disruptive, to return the focus to the person and their purpose for displaying them (Algase et al., 1996). Doing so may prevent these needs from being dismissed, overlooked, or ignored, resulting in increased quality of care and quality of life.

Increasingly acknowledged as better prognostic predictors of the worst outcomes, an unmet need describes a problem for which an individual does not receive adequate assessment or intervention which could meet said need (Ferreira et al., 2016). It is well-known that unmet needs in this patient population are associated with a decreased quality of life; increased anxiety, depression, BPSD, and somatic disorders; increased distress and dissatisfaction with services; premature institutionalization and mortality (Ferreira et al., 2016).

In 2013 Black and colleagues conducted a cross-sectional study of 254 community-residing PWD to determine the prevalence and types of unmet needs. They found that 99% of PWD had one or more unmet needs, of which the most common domains included “personal and home safety, general health and medical care, meaningful activities, legal issues and advance care planning, and evaluation and diagnosis of dementia” (Black et al., 2013, p. 2090).

Ferreira et al. (2016) conducted a cross-sectional study to explore the relationship between BPSD and unmet needs. They also aimed to describe which unmet needs contributed to BPSD in their sample of 166 nursing home residents with BPSD. They used the widely utilized psychometric of unmet needs, the Camberwell Assessment of Need for the Elderly (CANE) (Ferreira et al., 2016; Michelet et al., 2021). Consistent with prior studies, they found a high number of unmet needs which contribute to BPSD, including the absence of daytime activities and company (Ferreira et al., 2016; Michelet et al., 2021). Significant correlations between unmet and global needs and depressive symptoms, behavioral and psychological symptoms, and functional impairment were demonstrated (Ferreira et al., 2016). Based on these and prior findings, they concluded that the importance of identifying unmet needs is pivotal in the prevention and treatment of BPSD (Ferreira et al., 2016). A similar study by Michelet et al. (2021) of 451 PWD, also using CANE, found unmet needs for daytime activities and company were associated with increased affective and psychotic symptoms. They also suggest that interventions to address unmet needs for daytime activities and company may alleviate BPSD in PWD (Michelet et al., 2021).

While these cross-sectional studies cannot confer causality, they utilized validated measures of assessment that call attention to the prevalence of unmet needs. Due to the unfortunate physical and cognitive deterioration in late stages of ADRD, it is possible that unmet needs may actually be underrepresented in PWD in later stages who may not have been recruited. In addition to challenges to randomization, this represents a challenge to generalizability, yet reinforces the importance of active recognition on unmet needs in this patient population.

### Language and Attitudes Contributing to Unmet Needs

Perceptions, more aptly, misperceptions can increase the likelihood of unmet needs in PWD, with commensurate increases in BPSD due to deficient or maladaptive communication. Initially developed to not only enhance awareness of the lived experience of PWD, the term “BPSD” was also an attempt to phase out demeaning terminology such as “challenging behaviors” (Cunningham et al., 2019). However, in recent years, much controversy has emerged surrounding the usage of, and stigma associated with, the term “BPSD” as both label and assessment tool. In a similar vein of reframing behaviors as communication attempts of unmet needs, researchers (e.g., Dupuis et al., 2012; Macaulay, 2018; Cunningham et al., 2019) have also reexamined the utility and possible detriment of viewing these behaviors through a biomedical lens. The observations and concerns of these researchers reflect the tendency to pathologize behaviors, as opposed to understanding their true motivations, needs, and opportunities to intervene (Dupuis et al., 2012; Macaulay, 2018; Cunningham et al., 2019).

Dupuis et al. (2012) assert that the utilization of deficit-based and problem-based approaches ultimately pathologize and stigmatize behavior of PWD, leading to unnecessary suffering. They conducted an interpreted grounded theory study utilizing recorded interviews of 48 staff members in 18 long-term care facilities and elucidated the tendency for staff to filter behavior through the lens of pathology (Dupuis et al., 2012). They found that this pathology-based lens influenced how staff assigned meaning and characterization of behaviors as “challenging,” and consequently reacted *via* crisis management without consideration of underlying meaning (Dupuis et al., 2012). They further explained how PWD are thereby defined by their misinterpreted behaviors (i.e., “the wanderer,” “the screamer,” “the non-compliant”), despite the legitimacy of their behaviors considering their circumstances. Consequently, individuals are seen as a “burden” for both informal and formal caregivers, devaluing their humanity and dismissing their needs (Dupuis et al., 2012). This study highlighted the impact biomedical discourse may have on an individual’s quality of daily life, underscoring the value of understanding the meanings of actions through multiple perspectives (Dupuis et al., 2012).

Cunningham et al. (2019) conducted a literature review of criticisms and concerns of using BPSD to describe “normal human expressions in response to unmet needs” (Cunningham et al., 2019, p. 1110). Numerous movements and experts in the field have embodied this goal of prioritizing the individual, their personhood, and root causes of behaviors. A recent campaign, so named *#BanBPSD*, has gained momentum in recent years with the intention of improving awareness and care of PWD (Cunningham et al., 2019). Several issues of concern are asserted including chemical restraint by overmedication, prejudicial labeling, and breaches of human rights (Cunningham et al., 2019). Although there has been a growing interest in non-pharmacological approaches for BPSD, the overdependency on, and inappropriate use of pharmacological intervention is a major concern in this patient population, especially considering the increased risk of falls, stroke, death, and their small effect sizes (Cunningham et al., 2019).

Another noteworthy concern involves the premise that not all behaviors, including those associated with BPSD, are the direct result of neurological deterioration (Cunningham et al., 2019). An unpublished analysis conducted by the national Dementia Support Australia service in 2018 examined 3,566 cases and identified 50 factors contributing to behaviors across biological, social, and environmental domains (Cunningham et al., 2019). The most common contributing factors included pain (47%), carer approach (34%), and over or under stimulation, validating the importance of considering non-cognitive impairment factors experienced by PWD (Cunningham et al., 2019). A qualitative case study conducted by Macaulay (2018) involved her mother with AD living at a long-term care facility. Her detailed study of her mother’s treatment, staff behaviors and attitudes, and staff reporting echoed the concerns and observations experienced by many professionals and caregivers in the field of ADRD. Naomi Feil, founder of the Validation Method, also stressed the necessity to understand the person, in their entirety, and identify their needs, as “behavior cannot be judged appropriate or inappropriate unless it is viewed within the context of needs” (Macaulay, 2018, p. 178). The gross misrepresentations she observed directly, and reflected in medical notes, are one of an inordinate number of examples in the literature, thus reemphasizing the need to reframe BPSD in the context of PCC to maintain quality of life, well-being, and dignity for these individuals (Macaulay, 2018).

The salient endeavors of those who hold valid concerns regarding the BPSD paradigm are based upon the damage which ensues by inadequate efforts to understand the PWD. Similarly, they support the appreciation of the meanings underlying behaviors and communication attempts, which if done, may dramatically reduce distress and improve quality of life for these individuals (Cunningham et al., 2019). Concerns regarding the gravity of implications behind labels used with PWD are consistent with PCC in this regard. These studies could be limited by potential emotional bias, that may dually serve as a strength. Direct observation of the behaviors and interactions between carers and PWD through a research lens offers an opportunity for medicine and psychology to intersect. The qualitative data collected lend a perspective of humanity and a lesson of humility for carers and providers for interactions with PWD.

### Non-pharmacological Interventions

The needs-driven models previously discussed explain BPSD as attempts to communicate unmet needs and emphasize the significance of understanding. To this end, these models support the prodigious value of non-pharmacological interventions to manage BPSD. In fact, the most effective intervention for BPSD is training of formal caregivers (Bessey and Walaszek, 2019). Largely contraindicated for the treatment of BPSD, antipsychotic and other psychotropic have limited evidence for efficacy, and should be reserved for the most severe emergency situations due to their high risk of myocardial infarction, stroke, and mortality (Scales et al., 2018). Recent regulations state antipsychotic medications should only be considered “when the symptoms present a danger, and only after medical, physical, functional, psychological, emotional, psychiatric, social, and environmental causes have been identified an addressed” (Scales et al., 2018, p. 89).

A systematic review of the literature by Scales et al. (2018) from January 2010 to January 2017 found several non-pharmacological practices to address BPSD. Approaches supported by the literature included “sensory practices (aromatherapy, massage, multi-sensory stimulation, bright light therapy), psychosocial practices (validation therapy, reminiscence therapy, music therapy, pet therapy, meaningful activities), and structured protocols (bathing, mouth care)” (Scales et al., 2018, p. 88). These practices are person-centered, non-harmful, and require minimal to moderate effort and investment (Scales et al., 2018).

A literature review of 74 articles by Caspar et al. (2018) sought to identify elements of efficacy in non-pharmacological interventions for BPSD. Their results revealed some necessary components of successful outcomes including the caring environment, care skill development and maintenance, and the implementation of individualized care (Caspar et al., 2018). These constructs emphasize the importance of caregiver education and training, and consideration of both physical and social environmental factors affecting the PWD (Caspar et al., 2018).

Additionally, physical exercise in particular has been identified as a prominent intervention for BPSD and other symptoms of ADRD. de Souto Barreto et al. (2015) found physical activity reduced depression associated with BPSD, but not global BPSD in their systematic review and meta-analysis of randomized controlled trials (RTCs). A systematic literature review of mixed studies by Junge et al. (2020) on the effect of physical activity on BPSD also found positive effects of reducing BPSD, experiencing social rewards in group physical activities, the importance of walking outdoors, and PWD reported physical activity as means of maintaining personhood (Junge et al., 2020). The type, frequency, and intensity of exercise best suited to manage BPSD was not identified (de Souto Barreto et al., 2015), and may vary by individual health status.

Studies involving non-pharmacological interventions demonstrate significant support for several interventions. The challenge to real-world translation is such that the research is based on standardized protocols while asserting the importance of individualized person-centered methods. They do, however, provide a heuristic framework within which modification can be adapted.

## Discussion

The review of the literature identifies multidirectional relationships between the physical and psychosocial needs of PWD, difficulties and attempts to communicate said needs, and the potential misinterpretations of BPSD that result in these needs being overlooked, and therefore unmet. The stigma associated with cognitive decline, and the diagnosis of BPSD itself may further devalue the merit in purposeful understanding of behavior and communicative efforts. The sum of the literature indicates that at minimum, some manifestations of BPSD may in fact be attempts to communicate needs, and likewise frustrations regarding unmet needs, in PWD. This implores action to offer further education for providers and carers to view and treat PWD beyond their diagnosis.

The multitude of challenges inherent in studying this disease under the umbrella of understaffed, undertrained caregivers in institutions lacking adequate funding to provide the quality of care and safety intended for any person, represents a profound barrier in translating research to clinical practice. As such, the review of the literature to date poses many limitations, including challenges to generalizability, underrepresented populations, lack of long-term adoption of programs, difficulty maintaining education efforts, and the lack of individualized heuristic protocols. These obstacles are offset by the meaningful qualitative data obtained that offers a just, and humane lens through which to view and treat PWD. The intervention studies utilizing PCC approaches to the management of BPSD have elucidated the profound potential for meaningful communications and relationships ascertained by separating the disease and the human. Indeed, de-pathologizing behavior in this sense allows for the retention of dignity and personhood. The high prevalence of unmet needs demonstrated in the literature depicts a major gap in clinical care; a gap that can be bridged with minimal effort and investment, as evidenced by the aforementioned studies. Further research holds the potential to refine the data to identify best practices for carer education and program design, the positive clinical outcomes of which cannot be overstated in PWD and their caregivers.

## Conclusion

Behavioral and psychological symptoms of dementia create distress for both the person experiencing the symptoms, as well as caregivers and family members. The rich internal world of PWD can be difficult to ascertain due to deterioration of communication abilities, yet is ever-present, and should therefore be respected to the fullest degree. Taken together, the literature supports that BPSD may often be attempts to communicate feelings and thoughts of the PWD. When viewed through the lens of humanity, instances of “the water is too hot” or “I need to go to the bathroom” may appear as agitation or aggression; musculoskeletal pain or boredom may manifest as wandering. Appreciating BPSD as attempts to communicate, it is of utmost import for caregivers to facilitate understanding. Doing so may have far-reaching effects of improving the quality of life for PWD, as well as improving the communications and experience between PWD and caregivers. Caregiver awareness and training is sorely lacking at present, particularly with regard to communication approaches to BPSD. Further studies involving a systematized education program for caregivers that is easily accessible, if not ubiquitous, is sorely needed for this patient population and their caregivers. Furthermore, future studies could shed light on the efficacy of such education to reduce the stigma associated with cognitive decline. As pervasive and detrimental BPSD may be as a label or stigma, reframing it in this way may have profoundly positive effects for all those affected by ADRD. Reevaluating BPSD as a communication strategy may be challenging, but the benefits far outweigh the efforts, and allow PWD to retain their personhood and dignity.

## Author Contributions

The author confirms being the sole contributor of this work and has approved it for publication.

## Conflict of Interest

The author declares that the research was conducted in the absence of any commercial or financial relationships that could be construed as a potential conflict of interest.

## Publisher’s Note

All claims expressed in this article are solely those of the authors and do not necessarily represent those of their affiliated organizations, or those of the publisher, the editors and the reviewers. Any product that may be evaluated in this article, or claim that may be made by its manufacturer, is not guaranteed or endorsed by the publisher.
